# The role of allogeneic stem cell transplantation in the management of acute myeloid leukaemia: a triumph of hope and experience

**DOI:** 10.1111/bjh.16355

**Published:** 2019-12-10

**Authors:** Justin Loke, Ram Malladi, Paul Moss, Charles Craddock

**Affiliations:** ^1^ Centre for Clinical Haematology Queen Elizabeth Hospital Birmingham UK; ^2^ University of Birmingham Birmingham UK

**Keywords:** acute myeloid leukaemia, allogeneic stem cell transplantation, graft‐versus‐host disease, graft‐versus‐leukaemia

## Abstract

Acute myeloid leukaemia (AML) is the commonest indication for allogeneic stem cell transplantation (allo‐SCT) worldwide. The accumulated experience of allografting in AML over the last four decades has provided critical insights into both the contribution of the conditioning regimen and the graft‐versus‐leukaemia effect to the curative potential of the most common form of immunotherapy utilised in standard clinical practice. Coupled with advances in donor availability and transplant technologies, this has resulted in allo‐SCT becoming an important treatment modality for the majority of adults with high‐risk AML. At the same time, advances in genomic classification, coupled with progress in the accurate quantification of measurable residual disease, have increased the precision with which allo‐mandatory patients can be identified, whilst simultaneously permitting accurate identification of those patients who can be spared the toxicity of an allograft. Despite this progress, disease recurrence still remains a major cause of transplant failure and AML has served as a paradigm for the development of strategies to reduce the risk of relapse ‒ notably the novel concept of post‐transplant maintenance, utilising pharmacological or cellular therapies.

The number of adults allografted for acute myeloid leukaemia (AML) has risen sharply in the last decade (Passweg et al., 2019) – a reflection of both the limitations of current intensive chemotherapy (IC) regimens and also advances in transplant technology which have increased donor availability and reduced transplant toxicity. As a consequence, allogeneic stem cell transplantation (allo‐SCT) now plays a central role in the management of adults with AML in first complete remission (CR1). A number of factors have hitherto limited the optimal deployment of allo‐SCT in adults with AML. Firstly, identification of CR1 patients whose outcome will be poor if treated with IC alone who may consequently benefit from an allograft’s augmented anti‐leukaemic activity has been imprecise. In recent years, the cytogenetic and molecular risk stratification of AML (Dohner et al., 2017), coupled with early assessments of measurable residual disease (MRD) (Schuurhuis et al., 2018), has substantially improved our ability to identify allo‐mandatory patients. Secondly, the advent of novel stem cell sources for patients lacking a matched sibling or unrelated adult donor, notably cord blood and haploidentical donors, means that almost all eligible patients now have a potential donor. Thirdly, the demonstration that reduced intensity conditioning (RIC) regimens ameliorate transplant toxicity, has dramatically increased the number of transplant‐eligible patients. As a result, allo‐SCT can now be delivered with acceptable toxicity in many fit adults ≤75 years (Ringdén et al., 2019). Nevertheless, despite these advances, allo‐SCT still fails to deliver long‐term survival in a substantial proportion of patients. Although transplant toxicity has fallen significantly in recent decades (Gooley et al., 2010), the risk of disease recurrence has remained stubbornly high and novel strategies with the potential to reduce relapse risk are urgently required.

## Allo‐SCT in AML: a historical perspective

The first allogeneic transplant was performed for acute leukaemia in 1957 (Thomas et al., 1957). Outcomes improved steadily thereafter, consequent upon advances in supportive care, graft‐versus‐host disease (GVHD) prophylaxis, and crucially, HLA (human leucocyte antigen) typing (Thomas et al., 1975). Landmark randomised trials in the 1990s demonstrated that the intensity of the conditioning regimen was a critical determinant of relapse risk in the setting of a myeloablative conditioning regimen (MAC) (Clift et al., 1990, 1991). In addition to the important role of the conditioning regimen in disease control, the contemporaneous observation of an inverse correlation between relapse risk and the presence of GVHD identified the importance of the graft‐versus‐leukaemia (GVL) effect in the delivery of a durable anti‐tumour effect (Weiden et al., 1979). Subsequent studies have demonstrated the rapid emergence of a potenta and highly manipulable, GVL effect within the first three weeks post‐transplant in patients allografted for AML (Bacigalupo et al., 2000; Craddock et al., 2010).

The initial demonstration that allo‐SCT improved outcome in adults with AML in CR1 came from studies using a ‘donor versus no donor’ methodology, which demonstrated showing improved disease‐free survival and overall survival in patients with intermediate or adverse risk cytogenetics who had an available matched sibling donor (Cornelissen et al., 2007; Koreth et al., 2009). Critically, this survival advantage was only observed in patients under the age of 40 because of the toxicity of MAC regimens in older patients. In contrast, for patients with good risk cytogenetics and a low predicted risk of relapse when treated with chemotherapy alone, studies failed to demonstrate improved overall survival after allo‐SCT, since any reduction in relapse risk is offset by non‐relapse mortality (NRM) (Schlenk et al., 2008). The recent Recognition of the potency of the GVL effect led to the subsequent development of RIC regimens, which in the last 20 years have transformed transplant practice, permitting delivery of a potentially curative GVL effect in large numbers of older patients with AML, previously deemed too old for a transplant (Giralt et al., 1997; Slavin et al., 1998).

## Mechanisms and optimisation of a GVL effect

**Figure 1:**
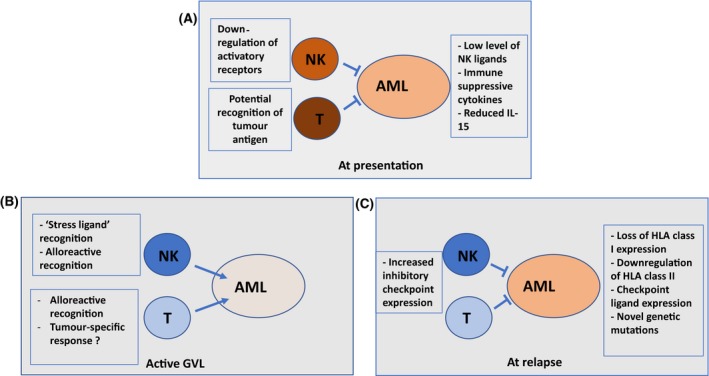
Representation of mechanisms determining immune recognition of acute myeloid leukaemia tumour cells by NK and T cells (A) at the time of disease presentation, (B) during a graft‐versus‐leukaemia response and (C) at the time of disease relapse after transplant.

It is now well‐established that the donor immune system can mediate a powerful GVL effect in many haematological malignancies. A general consensus has been that this is mediated primarily through an alloreactive T cell response, although there is increasing evidence that other mechanisms are also in operation. In order to assess potential mechanisms of GVL it is instructive to reflect on recent understanding of how AML can evade the immune system at the time of initial disease presentation (Fig 1A). The innate and adaptive arms of the immune response are both important in tumour‐specific responses ‒ a striking observation is that NK cell dysfunction is present in almost all patients at diagnosis (Fauriat et al., 2007). A range of mechanisms are observed, including down‐regulation of activatory receptors on NK cells, reduction of NK ligands on the tumour and decreased production of the potent NK‐stimulatory cytokine IL‐15, secondary to acquisition of the *FLT3* mutation (Mathew et al., 2018). The observation that leukemic stem cell populations often lack expression of NKG2D ligands, and are thus able to evade NK surveillance, represents one potential mechanism for tumour progression (Paczulla et al., 2019). Conversely, less is known regarding the importance of adaptive T cell immune responses during AML development, although high‐level expression of inhibitory checkpoint proteins and increased proportions of T‐regulatory cells are observed at diagnosis (Williams et al., 2019).

Graft‐versus‐leukaemia may potentially be mediated by both tumour‐specific and allo‐reactive recognition (Fig 1B). The infusion of innate cells within the donor graft may be capable of mediating an immediate tumour‐specific response and a recent analysis demonstrated relapse rates of 43% or 5% respectively for AML patients who received a donor stem cell infusion with an NK cell count that was either below or above the median cohort value (Maggs et al., 2017). This NK‐associated protection was most strongly correlated with the infusion of DNAM + cytotoxic subsets. The potential importance of NK‐mediated GVL is further shown through the correlation between a reduced rate of relapse, and both post‐transplant NK reconstitution within bone marrow as well as the use of a donor with an ‘activatory’ KIR B genotype (Cooley et al., 2009). The contribution of an adaptive immune responses against tumour‐specific targets is less clear, and although T cell recognition of proteins such as WT1 or PRAME can develop, these are typically of low frequency. Interestingly, the recent identification of high affinity antibody responses against an AML‐associated protein after SCT indicates a potential hitherto neglected importance for humoural immunity during GVL (van Balen et al., 2018).

Notwithstanding potential tumour‐specific immune responses, alloreactive recognition is clearly critical for disease control. NK cells which have a KIR‐ligand mismatch can mediate strong alloreactive responses, and epidemiological and laboratory studies have demonstrated the importance of this mechanism (Ruggeri et al., 2002). Indeed, myeloid cells are uniquely sensitive to the activity of alloreactive NK recognition and this may underlie some of the unique epidemiological features of GVL responses in AML patients. Nonetheless, alloreactive CD4^+^ and CD8^+^ T cell recognition of recipient minor histocompatibility antigens is thought to be the primary effector mechanism of GVL. This response may also manifest as GVHD but the tissue‐specificity of allo‐recognition, as well as subtle differences in the intensity and breadth of the T cell response, are thought to be important in determining clinical outcome (van Bergen et al., 2017).

Further insights into the GVL response are now starting to emerge through detailed studies of the mechanisms by which AML can relapse after transplant (Fig 1C). Importantly, these are largely determined by the acquisition of immune evasion by the tumour and again highlight the centrality of the GVL response in tumour control. Mechanisms include deletion of HLA class I genes, and downregulation of NK cell targets, together with increased expression of inhibitory checkpoint ligands and downregulation of HLA class II expression (Vago et al., 2009; Christopher et al., 2018; Jan et al., 2019; Toffalori et al., 2019). This latter point is of particular interest, giving emerging interest in the potential importance of CD4^+^ tumour‐specific responses in a wide range of malignant disorders.

These observations should now be translated into clinical strategies to prevent and treat relapse relapse. Novel potential approaches could include infusion of increased numbers of NK cells at the time of stem cell donation and optimisation of tumour and allo‐specific responses through appropriate donor selection, vaccination or immune modulation. Chimerism status will be a critical determinant of immune recognition and low levels of donor T cell engraftment are associated with increased disease relapse in some, but not all, studies (Wong et al., 2017). Donor lymphocyte infusion (DLI) is ineffective in overt morphological relapse where antigen‐specific cell therapy, including chimeric antigen receptor T (CAR‐T) cells, recognising AML‐associated proteins show preliminary promise. Checkpoint blockade has modest efficacy in this setting and carries a risk of graft‐versus‐host disease (GVHD) (Davids et al., 2016; Daver et al., 2018).

## Stem cell source and alternative donors

More than 30 million adults are now registered as potential volunteer donors on a range of national registries across the world, substantially increasing transplant options for patients lacking a matched sibling donor (MSD). Coupled with increased availability of good quality cryopreserved umbilical cord blood units and evidence that transplantation of haploidentical stem cells can now be achieved with acceptable toxicity, allo‐SCT can now be contemplated in almost all eligible patients. Unrelated adult donors and recipients are routinely typed by high resolution typing at HLA loci A, B, C, *DRB1* and *DQB1*. Broadly equivalent clinical outcomes are observed in patients with AML transplanted, using a well‐matched (8/8 or 10/10) unrelated volunteer donor (VUD), compared to those achieved with an MSD (Yakoub‐Agha et al., 2006; Gupta et al., 2010; Schlenk et al., 2010) In contrast, the use of a VUD donor with a one‐antigen mismatch is consistently associated with decreased overall survival, consequent upon an increased NRM and higher rates of acute and chronic GVHD (Verneris et al., 2015; Ayuk et al., 2018). Progressive levels of HLA mismatch further reduce overall survival and are not routinely recommended (Kollman et al., 2016; Ayuk et al., 2018), but may be modulated in the future by improvements in GVHD prophylaxis, such as the use of post‐transplant cyclophosphamide as a novel GVHD prophylaxis regimen (Battipaglia et al., 2019). The other major determinant of outcome after unrelated donor transplantation is donor age, with one study reporting a decrease in patient survival of 5% for every 10‐year increment in donor age (Kollman et al., 2016). As a consequence, clinicians are increasingly called on to decide whether to proceed with an older sibling donor in patients with AML, for whom a young well‐matched volunteer donor has also been identified. Current data are contradictory and although a CIBMTR study has favoured the use of an MSD (Alousi et al., 2013), a recent EBMT (European group for Blood and Marrow Transplantation) study in patients allografted for myelodysplasia showed a superior outcome using younger unrelated donors (Ayuk et al., 2013). An emerging factor complicating the choice of an older MSD is the possible influence of the presence of clonal haematopoiesis of indeterminate potential (CHIP) on transplant outcome. Interestingly, a large recent retrospective analysis observed that the presence of CHIP did not adversely impact survival and intriguingly was associated with a higher risk of chronic GVHD and lower risk of relapse (Frick et al., 2019). Prospective studies on the impact of the presence of CHIP on both transplant outcome and donor safety are now a priority.

The likelihood of identifying a suitably matched unrelated donor has risen to over 75% in Caucasian patients within the last decade (Gragert et al., 2014), but remains unacceptably low in some ethnic groups. The recent demonstration of encouraging transplant outcomes using single or double umbilical cord blood (UCB) units, matched at 4/6 or more HLA loci, is a major advance for patients lacking a suitably matched unrelated donor. Although associated with a modest increase in NRM, long‐term survival rates in patients transplanted using cord units of at least a 4/6 match with a high total nucleated cell dose are now comparable to those achieved using an MSD or well‐matched VUD in patients with AML transplanted using either a MAC or RIC conditioning regimen (Laughlin et al., 2001; Eapen et al., 2010; Brunstein et al., 2011). This reflects a steady reduction in the TRM (transplant‐related mortality) of cord blood transplants, but is also a possible reduction in relapse risk, particularly in patients with detectable MRD at the time of transplant (Milano et al., 2016; Shouval et al., 2019). Importantly, the toxicity of cord blood transplantation continues to fall, because of the meticulous selection of appropriately matched cord blood units with a high nucleated cell dose, the omission of ATG in GVHD prophylaxis, prompt treatment of the engraftment syndrome, and the use of letermovir to reduce the risk of CMV reactivation (Barker et al., 2017). As such, a cord blood transplant, using appropriately selected cord blood units, may possibly in future become the preferred donor source, even in patients with an available MSD, for selected patients with high‐risk AML.

More recently, it has been demonstrated that use of a HLA‐haploidentical family member can be associated with a modest NRM and acceptable risk of severe GVHD, if either post‐transplant ATG or cyclophosphamide is used as GVHD prophylaxis (Luznik et al., 2008; Lee et al., 2009). A number of retrospective registry studies show encouraging outcomes after haploidentical transplantation, using post‐transplant cyclophosphamide as GVHD prophylaxis, in adult AML. However, in the absence of prospective trials, the potential selection bias inevitable in registry analyses does not make it possible to confidently state whether a haploidentical donor should be preferred to a 9/10 or 10/10 unrelated adult donor (Piemontese et al., 2017). Two large recent EBMT studies have demonstrated continued incremental improvements in the outcome of patients with high‐risk AML transplanted using cord blood or haploidentical grafts, consistent with the possibility that selected patients with high‐risk AML may benefit from an alternative stem cell donor (Ruggeri et al., 2015; Versluis *et al*., 2017). The results from multi‐centre phase III randomised controlled trials of RIC SCT, which compare UCB or haploidentical donors (such as BMT CTN 1101; ClinicalTrials.gov Identifier: NCT01597778), are therefore awaited with interest, although it is noteworthy that transplant technologies have evolved during this period, which will make interpretation of such studies challenging.

## Towards a personalised conditioning regimen for allogeneic transplantation in AML

Conditioning regimens aim to deliver durable stem cell engraftment and maximal anti‐leukaemic activity, with minimal attendant toxicity. They are increasingly personalised to reflect patient‐ and donor‐specific characteristics. The ambition of achieving leukaemia‐free survival without the burden of significant GVHD is reflected in the increasingly adopted endpoint of ‘GVHD relapse‐free survival’ (GRFS) (Holtan et al., 2015). The comparative assessment of conditioning regimens, so essential to optimising transplant outcome, is complicated by the impact of patient and donor‐specific factors on both transplant toxicity and relapse risk. Specifically the risk of disease recurrence is determined by both tumour biology and pre‐transplant remission status, whilst transplant toxicity is determined by patient age, co‐morbidity and donor characteristics.

Until recently, conditioning regimens uniformly utilised myeloablative chemo‐radiotherapy, which imposes an absolute requirement for infusion of donor stem cells in order to avoid irreversible cytopenia. The development of reduced intensity regimens, which result in variable degrees of cytopenia, and by definition utilise ≤8 Gy TBI or ≤8 mg/kg busulfan (Bacigalupo et al., 2000), have dramatically increased the upper age limit for allo‐SCT and are now the commonest form of conditioning regimen deployed in adult AML allografts. Non‐myeloablative (NMA) regimens typically utilising fludarabine in combination with 2 Gy, do not require stem cell support, but are of limited utility in adult AML. The interpretation of recent randomised trials aimed at addressing the important question of whether a MAC or RIC regimen should be preferred in adults allografted for AML in CR1, have been complicated by striking regimen‐specific differences in toxicity and relapse associated with distinct MAC and RIC regimens (Scott et al., 2017; Fasslrinner et al., 2018). Future trials aimed at determining the optimal conditioning regimen in AML must therefore compare specific regimens and also incorporate both molecularly‐based disease risk as well as pre‐transplant embedded MRD assessment, which are such important determinants of relapse risk post‐transplant.

### What is the optimal MAC regimen in adult AML?

The MAC regimens most commonly used in AML have been combinations of cyclophosphamide (Cy) with either total body irradiation (TBI)‐ Cy/TBI‐ or busulfan (Bu)‐ Bu/Cy. Both regimens are still widely used in fit patients with high‐risk disease, transplanted using an MSD or VUD. The majority of studies comparing Cy/TBI with a Bu/Cy regimen (utilising oral Bu) have demonstrated broadly equivalent outcomes (Socie et al., 2001; Nagler et al., 2013). However, the advent of intravenous preparations of Bu associated with more predictable pharmacokinetics and an improved toxicity profile has reduced the toxicity of the Bu/Cy regimen; a prospective cohort study from the CIBMTR also demonstrated superiority of the iv‐Bu/Cy regimen, particularly for patients in CR1 (Bredeson et al., 2013). More recently, a large randomised trial compared the novel FluBu_4_ (12·8 mg/kg four days of IV busulfan) regimen with iv‐Bu/Cy for patients with AML in CR1. Whilst outcomes were similar between the groups, the FluBu_4_ regimen resulted in a significant reduction in NRM. As a consequence, Flu/iv‐BU is now an increasinly utilised MAC regimen in fit adults with AML in CR (Giralt, 2015), although irradiation‐based regimens still have an important role in the small number of patients presenting with either CNS involvement or an extramedullary sarcoma.

### What is the optimal RIC regimen in adult AML?

The optimal RIC regimen in older adults with AML has not been established. It is clear that Flu‐based regimens, typically formulated as Flu/Bu_2_ (6·4 mg/kg, two days of IV busulfan) (Slavin et al., 1998), Flu/melphalan (140 mg/m^2^) (Giralt et al., 1997) or Flu/Cy (1 g/m^2^ × 2) (Davies et al., 2018), differ in terms of both anti‐tumour activity and toxicity. There are regrettably very few randomised trials of RIC regimens in AML, but it is of interest that a randomised comparison between the widely‐used Flu/Bu_2_ regimen and the Seattle Flu/2 Gy TBI NMA regimen demonstrated equivalent overall survival, but an increased NRM and reduced relapse rate with the Flu/Bu_2_ regimen compared with the NMA regimen, underlining the importance of comparing specific reduced‐intensity regimens in the future (Blaise *et al*., 2013).

### Should a RIC regimen be preferred in all adults allografted for AML in CR1?

One of the most important, but still unresolved, questions in AML is whether RIC regimens should replace MAC regimens for all patients or remain restricted to an older age group, or those with significant co‐morbidities. Previous registry studies have demonstrated broadly equivalent outcomes in patients with AML, transplanted using an MAC or RIC regimen, noting that the lower NRM of a RIC regimen is offset by an increased risk of disease relapse. These retrospective studies have been difficult to apply to routine clinical practice because of the selection bias inherent within registry‐based studies and the heterogeneity of the MAC and RIC regimens included in these comparisons (Martino et al., 2006; Martino et al., 2013). Paradoxically, given the importance of the question, recruitment to practice‐informing prospective randomised trials comparing outcomes between MAC and RIC regimens has been challenging, but three have been reported in patients with AML or MDS (Bornhauser et al., 2012; Kroger et al., 2017; Scott et al., 2017; Fasslrinner et al., 2018). Two studies observed equivalent outcomes in patients transplanted using a busulfan‐based MAC or RIC regimen, but both studies recruited slowly and were consequently under‐powered (Kroger et al., 2017; Fasslrinner et al., 2018). In contrast, a US BMT Clinical Trials Network study observed improved disease‐free survival in patients transplanted using a MAC regimen, although survival outcomes were equivalent (Scott et al., 2017). Interpretation of this trial is, however, complicated by both an unexpectedly high relapse risk in patients allocated to receive an RIC regimen and very low relapse rate in the MAC arm. Critically, none of the randomised trials reported to date have incorporated pre‐transplant MRD evaluation which, coupled with disease biology, is now recognised as the most important predictor of relapse. On the basis of available data it is therefore not possible to support, with confidence, the conclusion that RIC regimens are superior to MAC regimens in adults with AML in CR1. Pragmatically, since the myeloablative Flu/Bu_4_ regimen can be delivered with acceptable rates of toxicity to most fit patients up to the age of 55 years, this should probably serve as the default regimen in AML CR1 patients, with an RIC regimen being preferred in older patients or those with significant co‐morbidities (Blaise et al., 2013; Malard et al., 2017).

## Which patients with AML in CR1 should proceed to transplant?

In order to optimise transplant outcome, it is vital that patients who are deemed likely to benefit from an allograft in CR1 should proceed to transplant as swiftly as possible. An important factor influencing the decision to allograft in CR1 concerns the wisdom of reserving transplants for patients who relapse and achieve a second CR with salvage chemotherapy. The outcome of relapsed AML remains poor, reflecting both the small chance of achieving a second CR after salvage chemotherapy in patients with an intermediate or adverse risk karyotype, and inferior transplant outcomes in patients allografted in second CR (Breems et al., 2005; Ganzel et al., 2018). Consequently, once it has been decided that a patient is likely to derive a significant survival benefit from allo‐SCT, it is unwise to postpone transplantation unless it is deemed that the patient has a high chance of achieving a second CR with salvage chemotherapy, or for reasons of personal considerations.

### The decision‐making process in CR1 patients

The pivotal question in deciding whether or not to recommend an allograft in CR1 is whether the reduction in relapse risk delivered by a transplant outweighs the attendant NRM. Studies show that, as a broad estimate, allo‐SCT halves the relapse risk for CR1 patients in comparison with chemotherapy, a finding evident in all cytogenetic risk groups (Cornelissen et al., 2012a,b). As has been so insightfully articulated by Cornelissen *et al*., transplant decisions should therefore be made on the basis of an individualised assessment of both the predicted risk of disease relapse, if patients are treated with chemotherapy alone, and the NRM, calculated on the basis of patient age, fitness and donor source. Since the NRM of fit adults transplanted using a matched sibling or unrelated donor can be reliably predicted to be in the region of 15%, patients with a relapse risk >50% are likely to benefit from the attendant halving of relapse risk delivered by an allograft, using a well‐matched donor. Furthermore, patients with a higher risk of disease relapse can be reasonably considered as transplant candidates, even when the predicted NRM is higher on account of patient co‐morbidities or increased patient:donor HLA disparity. By similar reasoning, patients with a relapse risk ≤40% after chemotherapy are very unlikely to derive any survival advantage from an allograft, given the small but finite risk of transplantation, even in the most favourable of circumstances (Table 1).

**Table 1:** Selection of patients with acute myeloid leukaemia in first complete remission for allogeneic stem cell transplantation (allo‐SCT), based on relapse risk (Döhner et al., 2017; Schuurhuis et al., 2018) and estimate of non‐relapse mortality (NRM) (Sorror et al., 2014), adapted from Cornelissen and Blaise (2016).

2017 ELN Risk stratifications by genetics	MRD after cycle 2 chemotherapy	Estimated risk of relapse, based on consolidation with:		Maximal tolerated NRM prognostic scores for allo‐SCT to be beneficial	
		Chemotherapy alone (%)	Allo‐SCT (%)	HCT‐CI score	NRM risk (%)
Favourable	Negative	25–35	15–20	N/A (<1)	5
	Positive	70–80	30–40	≤3–4	<30
Intermediate	Negative	50–60	25–30	≤2	<20
	Positive	70–80	30–40	≤3–4	<30
Adverse	N/A	>90	45–55	<5	<35

### Risk stratification in patients treated with IC alone

The last decade has seen major progress in predicting the risk of disease relapse in adults with AML treated with IC. Factors associated with a higher risk of disease relapse include increased patient age, a high white blood cell count at presentation, a diagnosis of secondary AML, the presentation karyotype, and the delayed acquisition of morphological remission (Dohner et al., 2017). More recently, the development of a genomic classification of AML, based on the identification of mutations in transcription factors, epigenetic modifiers, spliceosome, cohesin complex, and signaling pathways (Papaemmanuil et al., 2016) have underpinned the development of more accurate risk stratification models. These have been encapsulated in the 2017 ELN classification, which now includes presentation karyotype and mutations in *NPM1*, *FLT3* and *CEBPA*, *RUNX1*, *ASXL1* and *TP53* genes to provide a genetic risk stratification of AML (Dohner et al., 2017). In the future, we can anticipate the greater precision in outcome prediction provided by prospective evaluation of large genomically characterised cohorts treated with IC alone, as well as hopefully with allo‐SCT, to result in further refinement of such models (Gerstung et al., 2017).

**Figure 2:**
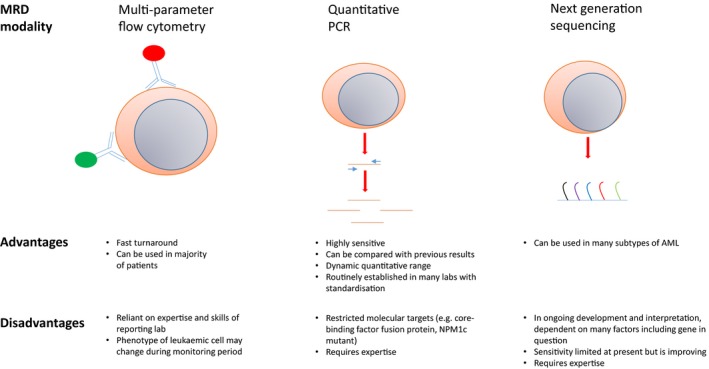
Measurable residual disease measurement methods in acute myeloid leukaemia.

Immunophenotypic or molecular quantification of MRD after induction or consolidation therapy also provides an important independent predictor of relapse risk in AML and can be used to refine conventional morphological assessment of response (Terwijn et al., 2013; Ivey et al., 2016; Freeman et al., 2018) (Fig 2). More recently, next generation sequencing technologies have been shown to improve risk stratification in combination with conventional MRD methodologies, incorporating strategies to exclude mutations in genes associated with age‐related clonal hematopoiesis (Jongen‐Lavrencic et al., 2018). When used for risk stratification, it is important to note that the specific impact of MRD status on relapse risk is critically dependent on patient age, cytogenetic and molecular subgroup, and time of assessment.

### Predicting transplant outcome – the role of donor source and patient co‐morbidities

The critical determinants of transplant‐related death after allo‐SCT for AML are patient age and fitness, the degree of HLA disparity and conditioning regimen intensity. Two scoring systems have been shown to predict transplant outcome, specifically NRM, in the setting of allo‐SCT for AML. The EBMT score was the first to demonstrate that a scoring system which captured age, donor:HLA disparity and disease status was able to identify patient populations whose NRM ranged from 15% ‒ 45% (Gratwohl et al., 2009). The HCT‐CI, derived using a weighted assessment of 17 co‐morbidities, has also been shown to predict NRM and outcome after allo‐SCT and has been validated in AML (Sorror et al., 2007). Predicting the impact of patient fitness on transplant outcome is however still imprecise, particularly in older patients, and the ability of a machine‐based learning model to increase the accuracy of prediction therefore represents a potentially important advance (Shouval et al., 2015). In the meantime, it remains a fact that one of the most challenging aspects of daily transplant practice is the accurate identification of older patients with marginal co‐morbidities, for whom the varied toxicities of transplant represent an acceptable risk ‒ to both patient and physician.

### The role of allo‐SCT in the management of AML beyond CR1

Whilst a small proportion of patients with AML in second CR achieve long‐term survival if treated with salvage chemotherapy alone, compelling data identify allogeneic transplantation as the preferred curative option (Gale et al., 1996). Long‐term survival rates in the region of 30–50% have been reported after transplantation from either a matched sibling or unrelated adult donor (Tauro et al., 2005; Gilleece et al., 2019). Encouraging results are also reported in patients transplanted with alternative donors. In patients who have achieved a morphological second CR, it is wise to proceed immediately to transplant, providing the patient is fit, a donor has been identified, and there is no evidence supporting further courses of chemotherapy prior to transplantation. Whilst a rigorous comparison of MAC and RIC regimens has not been performed in fit patients <50 years, a MAC regimen should probably be preferred.

Ten to 40 per cent of adults with newly diagnosed AML fail to achieve a morphological complete remission (CR) after two courses of induction chemotherapy (Ferguson et al., 2016) and are therefore defined as primary refractory AML (PREF AML; Dohner et al., 2017). Evidence that allo‐SCT can deliver long‐term survival in a significant proportion of patients with PREF AML has been accumulating over the last decade and represents an important advance for this sizeable patient population for whom no other effective therapy exists (Duval et al., 2010; Ferguson et al., 2016). The optimal conditioning regimen in patients with PREF AML remains a matter of conjecture. Whilst MAC regimens should probably be preferred in fit patients <50 years, encouraging results have also been reported using the sequential FLAMSA regimen which incorporates additional tumour debulking with cytosine arabinoside and amsacrine, prior to a Flu‐based RIC regimen (Schmid et al., 2006).

### Considerations for elderly patients with AML

The incidence of AML rises sharply with age. Particular challenges associated with the management of older patients with AML include the increased proportion of patients with adverse cytogenetic or molecular characteristics, as well as challenges in assessment of co‐morbidities and fitness for intensive therapies (Schiffer et al., 1989). Furthermore, clinicians have traditionally been more reticent to refer elderly patients for allo‐SCT (Estey et al., 2007), despite emerging evidence that for a proportion, at least, of older patients, allografting represents an important potentially curative treatment modality. RIC conditioning regimens have resulted in an increase in the proportion of allo‐SCT patients >70 years who are transplanted (Muffly et al., 2017) with acceptable two‐year overall survival rates in a large EBMT registry cohort (Ringdén et al., 2019). A prospective phase II trial demonstrated particularly encouraging results, confirming that an RIC regimen can safely be delivered to patients aged 60–74 years old with AML in CR1 (Devine et al., 2015). There is some debate as to how much age contributes to transplant risk, with some suggestion that it is limited in isolation (McClune et al., 2010; Sorror et al., 2011; Chevallier et al., 2012).

A fundamental challenge in optimising transplant management of older patients remains the identification of those who can safely tolerate an allograft. At present, the selection of patients is based on a co‐morbidity scoring performed prior to transplantation. The most commonly used co‐morbidity score, the HCT‐CI (Sorror et al., 2005), was established for patients with a median age of 44. A subsequent update of this model to include older patients (Sorror et al., 2014) has been shown to be of particular value in transplant decision‐making for patients >60 years, in whom the NRM of patients with a HCT‐CI of 0‐1 is broadly similar to that observed in younger patients, in contrast to the NRM of 60% observed in patients with a score of 2 or greater (Nikolousis et al., 2015). An additional comprehensive geriatric assessment tool has been developed to take into account a more sophisticated functional status of patients, prior to transplantation (Muffly et al., 2013).

## Reducing relapse risk

**Figure 3:**
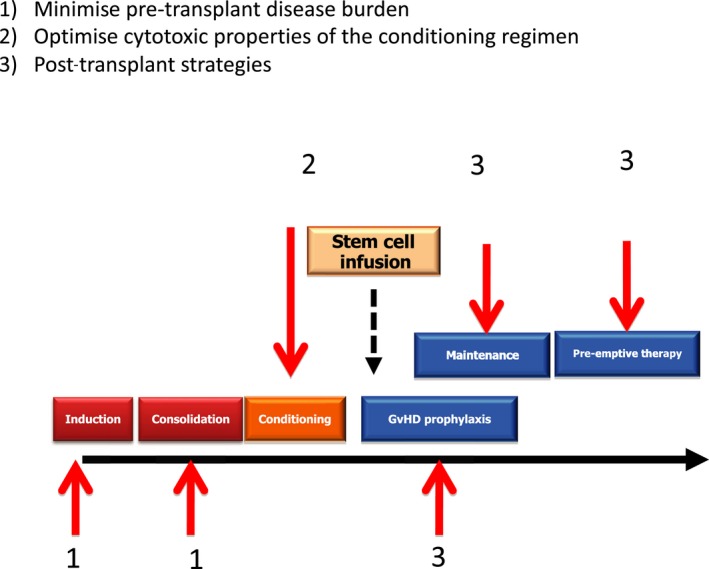
Strategies to reduce the risk of disease relapse in patients allografted for acute myeloid leukaemia.

Disease relapse remains the major cause of treatment failure in patients allografted for AML and ranges between 30% and 80% according to disease stage at transplant, presentation karyotype, and the intensity of post‐transplant immunosuppression (Bacigalupo et al., 1991; Tallman et al., 2007; Craddock et al., 2010, 2011). Increased understanding of the factors determining risk of relapse has permitted the development of a range of pre‐, peri‐ and post‐transplant strategies with the potential to reduce this problem (Fig 3). Broadly, these include reducing the disease load as assessed by MRD status prior to transplant, increasing conditioning regimen intensity without incurring additional toxicity, and maximising a GVL‐response post‐transplant.

### Reduction in pre‐transplant MRD status as a strategy to reduce the risk of relapse

A number of retrospective analyses have identified pre‐transplant MRD levels to be an important prognostic risk factor of relapse, although it should be noted that prospective studies are lacking (Buckley et al., 2017). A very important question is whether interventions aimed at reducing the pre‐transplant MRD burden can improve transplant outcome. Retrospective registry studies have failed to demonstrate that the number of cycles of IC delivered prior to either a MAC or RIC transplant can impact on relapse risk, but importantly none of these studies included MRD assessment (Tallman et al., 2000; Warlick et al., 2014). Two recent randomised trials examining the respective impact of midostaurin or CPX‐351, compared with DA induction chemotherapy, in addition to demonstrating improved survival in the experimental arm, also showed improved transplant outcome (Stone et al., 2017; Lancet et al., 2018). Neither study included MRD quantification and the reason for the improved outcome remains conjectural, but it is tempting to speculate that augmented induction chemotherapy was associated with a lower MRD load pre‐transplant. Consequently, there is an urgent need for prospective randomised studies which examine the impact of pre‐transplant therapeutic intervention on both pre‐transplant MRD status and relapse risk post‐allograft.

### Optimising the conditioning regimen

Given the central importance of the conditioning regimen in determining relapse risk, there remains an important requirement for randomised comparisons of both MAC and RIC regimens, particularly in high‐risk patients. Critical to the interpretation of such studies is a recognition that the anti‐tumour properties of both regimens are also critically impacted by GVHD prophylaxis strategy, including the intensity of post‐transplant immunosuppression and whether or not T cell depletion is utilised (Pasquini et al., 2012). Attempts to add sequential chemotherapy to RIC regimens, such as the FLAMSA schedule, has been reported to reduce disease relapse in high‐risk AML in single arm studies, and the results of a randomised comparison with the FB_2_ regimen performed by the UK NCRI AML Group is awaited (Schmid et al., 2005; Malard et al., 2017).

### Post‐transplant strategies to reduce transplant risk

**Figure 4:**
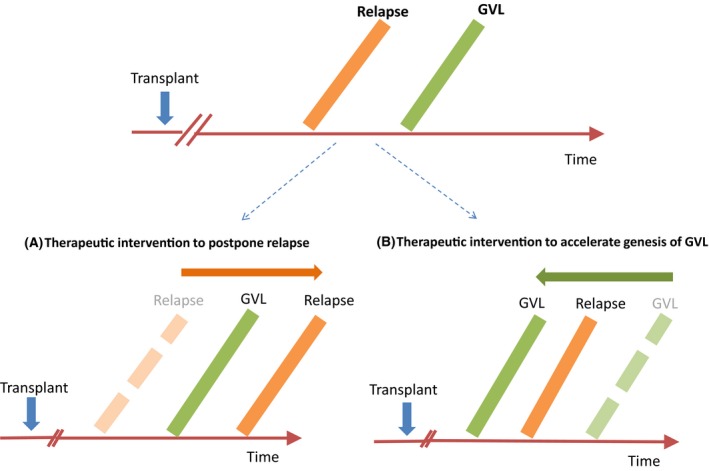
Post‐transplant maintenance strategies to reduce relapse risk in patients allografted for acute myeloid leukaemia. (A) Pharmacological acceleration of a graft‐versus‐leukaemia (GVL) effect; (B) pharmacological manipulation of the kinetics of disease relapse to ‘buy’ time for the genesis of a GVL effect.

The hypothesis that pharmacological intervention in the early post‐transplant period can manipulate the kinetics of disease relapse and may have the potential to improve transplant outcome was first established in chronic myeloid leukaemia using imatinib (Olavarria et al., 2007), and has been extended to patients allografted for AML and MDS (Craddock, 2013). Conceptually, there are a number of mechanisms by which adjunctive post‐transplant therapies might reduce the risk of disease recurrence (Fig 4). Firstly, drugs with inherent anti‐leukemic activity may simply augment the anti‐tumour activity of the transplant. Secondly, the manipulation of the kinetics of disease relapse may give the emerging allo‐immune effect a competitive advantage, buying time for the establishment of a clinically significant GVL effect, prior to disease relapse. Thirdly, postponement of disease relapse can in principle permit delaying the administration of DLI until such time that the risk of severe GVHD is reduced. Finally, post‐transplant pharmacological therapies may directly modify the allo‐reactive response, potentially through up‐regulation of tumour antigens (Choi et al., 2010; Goodyear et al., 2012) or acceleration of T cell reconstitution (Mathew et al., 2018).

One of the most promising post‐transplant maintenance strategies currently under examination is the administration of *FLT3* inhibitors in patients allografted for *FLT3 *ITD+ AML. A number of retrospective studies have demonstrated a reduced risk of disease relapse in those patients allografted for *FLT3* ITD+ AML, who received post‐transplant sorafenib maintenance (Brunner et al., 2016), but do not currently justify the routine administration of *FLT3* inhibitors in this patient group. The results of the US CTN1506 trial examining the impact of post‐transplant gilteritinib maintenance therapy in patients allografted for Flt3+ AML is therefore eagerly awaited (NCT02997202) (Levis et al., 2019).

An alternative and promising approach is peri‐ or post‐transplant administration of the DNMT inhibitors AZA or decitabine, which are also both well‐tolerated in this clinical setting (Cruijsen et al., 2016). Both agents have the capacity to induce CD8^+^ T cell responses to candidate tumour antigens, including MAGE antigens, which are not ordinarily observed post‐transplant, and prospective randomised trials of the impact of such agents as post‐transplant maintenance are ongoing (Craddock et al., 2016a).

## Management of relapsed AML post‐transplant

**Figure 5:**
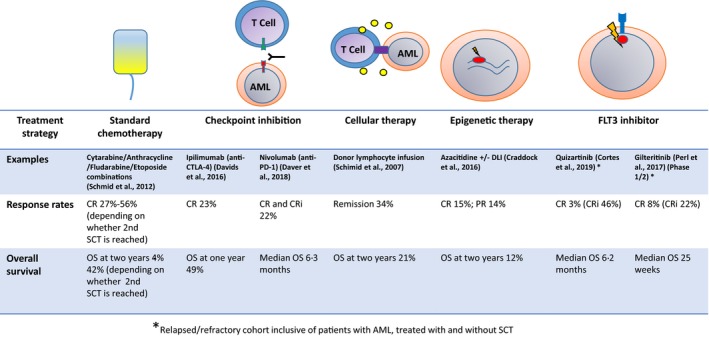
Management of relapse postallogeneic stem cell transplantation in acute myeloid leukaemia, including immune modulation and chemotherapy strategies.

The outcome for patients who relapse after allograft remains very poor, with a two‐year OS of 14–25%, although the small number of patients who achieve a second CR after salvage therapy can have a promising outcome after either a second allograft or DLI (Schmid et al., 2012, Christopeit et al., 2013) (Fig 5). Pivotal to the development of novel treatment strategies is the development of a better understanding of the biology of disease relapse post‐transplant.

It is increasingly recognised that clonal evolution is a fundamental mechanism underlying disease relapse post‐transplant (Quek et al., 2016; Jan et al., 2019). The observation that potentially druggable mutations, such as *FLT3* ITD, are commonly lost at relapse may be interpreted as supporting the exploration of maintenance strategies which utilise drug or cellular therapies with broader anti‐tumour specificity as opposed to targeted therapies. There is also increased evidence of acquired dysregulation between the donor immune system and recipient haematopoiesis as an important mechanism of disease relapse (Fig 1). Acquired uniparental isodisomy of the HLA genes on chromosome 6p, such that the haplo‐mismatched alleles are lost, is commonly observed in patients who relapse after a haploidentical transplant(Vago et al., 2009). A similar process, and direct HLA gene deletion, can occasionally be seen after VUD SCT (Toffalori et al., 2012; Hamdi et al., 2015; Jan et al., 2019) but appears to be a rare event after MSD. The recent discovery of transcriptional downregulation of HLA Class II expression in leukaemia cells at relapse demonstrates another means by which the GVL mechanism is circumvented (Toffalori et al., 2019).

A major challenge in the management of relapsed disease is the tolerability of salvage therapies in post‐transplant patients. Azacitidine has recently been shown to be a well‐tolerated and active salvage agent in patients relapsing post‐allograft (Craddock et al., 2016b), and may also be combined with DLI (Schroeder et al., 2015). A wave of novel agents have also shown promise. A recent trial of lenalidomide and azacitidine showed overall clinical responses of 47% in a phase I clinical trial (Craddock et al., 2019). Quizartinib and gilteritinib are active in patients with *FLT3* ITD+ disease and appear superior to high dose salvage chemotherapy (Perl et al., 2017; Cortes et al., 2019). An important question in coming years will be whether pre‐emptive interventions for MRD positive disease will be beneficial for patients. Novel immunotherapeutic strategies will be important, but many remain at either a pre‐clinical or early phase trial setting. The fundamental challenge is of identifying a target antigen that is expressed specifically and highly on AML cells (Perna et al., 2017). Bi‐specific antibodies (BiTE) directed against AML cells through either CD33 (Herrmann et al., 2018) or CD123 (Al‐Hussaini et al., 2016), can be brought into contact, and activate T cells, through CD3. CAR‐T have also been explored. Targets include CD33, CD123 and NKG2DL (Ritchie et al., 2013), and some early short‐lived responses, but results of further clinical trials are awaited.

## Future challenges

The last decade has seen a remarkable increase in the number of therapeutic opportunities with the potential to reduce the risk of disease relapse in patients allografted for AML. A future priority must be to effectively deliver randomised trials of transplant regimens and post‐transplant maintenance strategies, incorporating both genomic and pre‐transplant MRD studies. This will require far‐reaching improvements in our capability to deliver practice‐informing transplant trials. Much can be learned from the remarkable success of the accelerated trials network developed by the US BMT Clinical Trials Network (Network, 2016). Attention must also be given to the routine integration of genomics and biomarker discovery programmes as well as developing new models of partnership between academia and the pharmaceutical sector. By accelerating the delivery of practice informing trials to regulatory standards, such innovative networks will drive effective collaboration between the transplant community and the biopharmaceutical sector, to the ultimate benefit of patients.

## Declaration of Interest

CC has received honoraria from Celgene, Daichi‐Sankyo, Novartis and Pfizer, as well as research funding from Celgene. JL has received travel funding from Novartis and Daichi‐Sankyo.
